# Serum proteomics reveals APOE dependent and independent protein signatures in Alzheimer’s disease

**DOI:** 10.21203/rs.3.rs-3706206/v1

**Published:** 2024-01-09

**Authors:** Valborg Gudmundsdottir, Elisabet Frick, Valur Emilsson, Thorarinn Jonmundsson, Anna Steindorsdottir, Erik C.B. Johnson, Raquel Puerta, Eric Dammer, Anantharaman Shantaraman, Amanda Cano, Merce Boada, Sergi Valero, Pablo Garcia-Gonzalez, Elias Gudmundsson, Alexander Gudjonsson, Rebecca Pitts, Xiazi Qiu, Nancy Finkel, Joseph Loureiro, Anthony Orth, Nicholas Seyfried, Allan Levey, Agustín Ruiz, Thor Aspelund, Lori Jennings, Lenore Launer, Vilmundur Gudnason

**Affiliations:** University of Iceland; Icelandic Heart Association; Icelandic Heart Association; Icelandic Heart Association; University of Iceland; Emory University School of Medicine; Ace Alzheimer Center Barcelona; Emory University; Goizueta Alzheimer’s Disease Research Center; Ace Alzheimer Center Barcelona; Research Center and Memory Clinic of Fundació ACE, Institut Català de Neurociències Aplicades-UIC, Barcelona; Ace Alzheimer Center Barcelona; Ace Alzheimer Center Barcelona; Icelandic Heart Association; Icelandic Heart Association; Novartis Institutes for Biomedical Research; Novartis; Novartis Institutes for Biomedical Research; Novartis; Emory University School of Medicine; Emory; Fundació ACE; University of Iceland; Novartis; National Institute on Aging, National Institutes of Health; Icelandic Heart Association

**Keywords:** Alzheimer’s disease, APOE, Proteomics, Mendelian randomization, Longitudinal study, Cross-sectional study, CSF, Brain, Network

## Abstract

The current demand for early intervention, prevention, and treatment of late onset Alzheimer’s disease (LOAD) warrants deeper understanding of the underlying molecular processes which could contribute to biomarker and drug target discovery. Utilizing high-throughput proteomic measurements in serum from a prospective population-based cohort of older adults (n = 5,294), we identified 303 unique proteins associated with incident LOAD (median follow-up 12.8 years). Over 40% of these proteins were associated with LOAD *independently* of *APOE*-ε*4* carrier status. These proteins were implicated in neuronal processes and overlapped with protein signatures of LOAD in brain and cerebrospinal fluid. We found 17 proteins which LOAD-association was strongly *dependent* on *APOE*-ε*4* carrier status. Most of them showed consistent associations with LOAD in cerebrospinal fluid and a third had brain-specific gene expression. Remarkably, four proteins in this group (TBCA, ARL2, S100A13 and IRF6) were downregulated by *APOE*-ε*4* yet upregulated as a consequence of LOAD as determined in a bi-directional Mendelian randomization analysis, reflecting a potential response to the disease onset. Accordingly, the direct association of these proteins to LOAD was reversed upon *APOE*-ε*4* genotype adjustment, a finding which we replicate in an external cohort (n = 719). Our findings provide an insight into the dysregulated pathways that may lead to the development and early detection of LOAD, including those both independent and dependent on *APOE*-ε*4*. Importantly, many of the LOAD-associated proteins we find in the circulation have been found to be expressed - and have a direct link with AD - in brain tissue. Thus, the proteins identified here, and their upstream modulating pathways, provide a new source of circulating biomarker and therapeutic target candidates for LOAD.

## Introduction

Alzheimer’s disease (AD) is the most common cause of dementia, accounting for up to 80% of all dementia cases^1^, of which *late onset* Alzheimer’s disease (LOAD) is most common^2^. As of 2022, approximately 55 million individuals worldwide had dementia, representing 1 out of 9 people aged 65 and over^3^. While promising advances have been made in amyloid-targeting therapeutic options for early-stage LOAD^4,5^, they still have limited benefit and identification of additional risk pathways that can be used for early detection and intervention is highly needed. To meet these demands, a variety of biologically relevant circulating molecules have been broadly associated with LOAD risk. The proteome in particular has the potential to reveal circulating markers of disease-related molecular pathways from different tissues, and studies assessing the circulating proteomic signatures between non-demented older adults and individuals suffering from LOAD have been described^6–17^. Modest sample sizes, low-throughput proteomics and lack of longitudinal measurements have, however, been limiting factors in these studies. However, a recent large-scale longitudinal study identified promising blood-based markers for all-cause incident dementia although it is unknown how specific the results are to LOAD^18^. Information on the global circulating proteomic profile preceding the onset of LOAD, and how well it reflects AD-related processes in brain and CSF, is thus scarce.

Alzheimer’s disease has a considerable genetic component, and both common^19^ and rare risk variants have been identified^20^, of which the strongest effects are conferred by variants in the well-known apolipoprotein E (*APOE*) gene. Approximately 25% of the general population carries the *APOE*-ε*4* variant while it is present in over 50% of AD cases^21,22^. The *APOE*-ε*4* allele increases the risk of LOAD by threefold in heterozygous carriers and up to twelvefold in homozygous carriers^23^. Although the link between the ε*4* allele and LOAD has been extensively researched, light has yet to be shed on the precise mechanism by which the *APOE* gene affects LOAD onset and/or progression. Importantly, recent large-scale proteogenomic studies have consistently established the *APOE* locus as a protein-regulatory hotspot, regulating levels of hundreds of proteins in both circulation^24–27^ and cerebrospinal fluid (CSF)^28,29^. Yet, it remains unknown to what extent these proteins relate to LOAD and if they can provide new information on the mechanisms through which *APOE*-ε*4* mediates it risk. Identifying LOAD-associated circulatory proteins and whether their association is *APOE*-dependent or independent is crucial for the understanding of AD more generally as well as for gaining insight into potential pathways suitable for targeting in personalized treatment.

**Figure 1:**
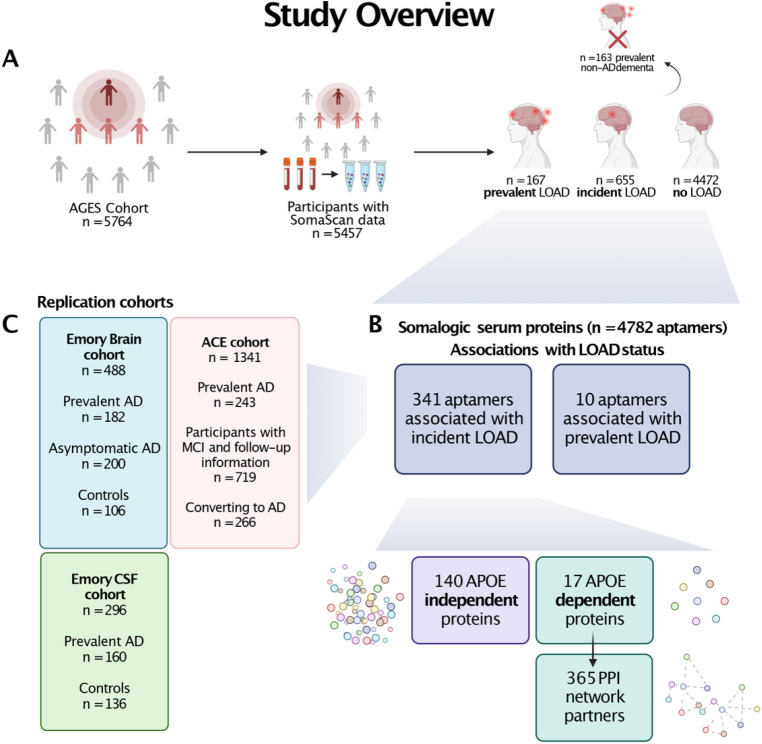
Study overview Flowchart of the current study. **a)** Overview of the AGES cohort and study participants. Prevalent non-AD dementia cases were excluded from the analysis. **b)** Overview of the aptamers tested and their associations with LOAD. Serum measurements of 4782 aptamers were associated to prevalent and incident LOAD status, using logistic and Cox proportional hazards regression models, respectively. From the proteins associated with incident LOAD, sets of 140 proteins with an *APOE*-independent associations and 17 proteins with an *APOE*-dependent association were defined. The *APOE*-dependent proteins were further expanded to first degree protein-protein interaction (PPI) partners. All sets of proteins were subjected to functional enrichment analysis and bi-directional Mendelian Randomization (MR) analysis. **c)** Overview of the replication cohorts used in the study which include proteins measured in the circulation (ACE) as well as in brain and CSF (Emory).

The current study tests the hypotheses that specific proteomic signatures in the circulation precede LOAD diagnosis and can reflect dysregulated biological pathways in the brain and CSF. Furthermore, we expect that some of these protein signatures may be affected by the *APOE-*ε*4* genotype and can thus provide molecular read-out of pathways directly affected by *APOE*-ε*4*. To address these hypotheses, we used a high throughput aptamer-based platform to characterize 4,137 serum proteins in 5,294 participants of the population-based Age, Gene/Environment Susceptibility-Reykjavik Study (AGES-RS)^30^ to identify protein signatures of incident LOAD (events occurring during follow-up) and prevalent LOAD, taking an unbiased, longitudinal, and cross-sectional approach to the discovery of potential biomarkers for LOAD (Fig. 1). Considering *APOE*’s protein-regulatory influence and how it may impact the way that serum-proteins are associated with LOAD, we disentangled the LOAD protein signature into *APOE*-ε*4* dependent and independent components, by identifying proteins whose LOAD-association is largely attenuated upon conditioning on *APOE*-ε*4* carrier status. We compared the serum protein signature of LOAD to those observed in cerebrospinal fluid (CSF) and brain and finally, used genetic variation as anchors to determine the potential causal direction between serum proteins and disease state.

## Results

### The AGES study cohort.

This prospective population-based study was based on 5,127 participants free of dementia at baseline, after the exclusion of 163 individuals with prevalent non-AD dementia, and 167 individuals with prevalent LOAD. During a potential follow-up of 12.8 years (median), 655 individuals were diagnosed with incident LOAD, with the last individual being diagnosed 16 years from baseline. Participants with incident LOAD were older at entry, were more likely to carry an *APOE*-ε*4* allele, had lower BMI, and had lower education levels compared to healthy individuals (Supplementary Table 1). See Fig. 1 for study overview.

### Serum protein profile of incident LOAD in AGES.

**Figure 2:**
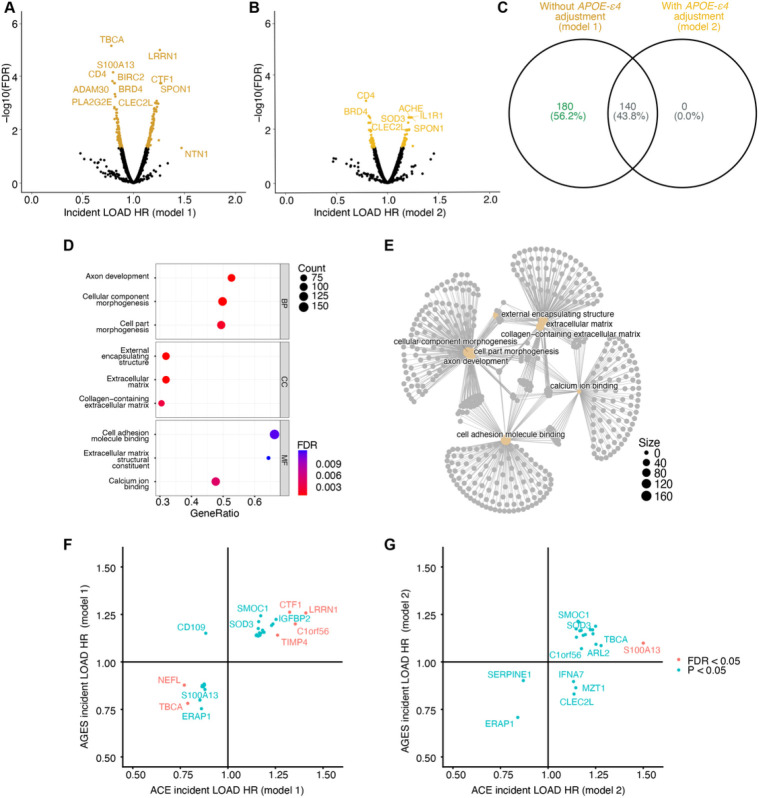
Proteins associated with incident LOAD in AGES (n = 5127). **a-b)** Volcano plots showing the protein association profile for incident LOAD from the Cox PH **a)**without *APOE*-ε*4* adjustment (model 1) and **b)** with *APOE*-ε*4* adjustment (model 2). **c)** Venn diagram for the overlap between models 1 and 2 for incident LOAD. **d-e)** Enrichment of top Gene Ontology terms from GSEA analysis for incident LOAD (model 1) shown as **d)** dotplot, stratified by ontology and **e)** gene-concept network. **f-g)** Comparison of effect sizes (HR) for incident LOAD between the AGES and the ACE (n = 719) cohorts for all proteins reaching nominal significance (P < 0.05) in the Cox PH in ACE for **f**) model 1 and **g**) model 2.

To investigate the LOAD-associated circulatory proteomic patterns which occur prior to disease onset, we used Cox proportional hazards (Cox PH) models and found 320 aptamers (303 proteins) to be significantly (FDR < 0.05) associated with incident LOAD diagnosis after adjusting for age and sex (model 1), with hazard ratios (HRs) ranging from 0.78 for TBCA to 1.47 for NTN1 (Fig. 2d, Supplementary Table 2). To account for variability related to *APOE*-ε*4* carrier status, we adjusted for the genotype in an additional model (model 2, Supplementary Table 2), which resulted in 140 significant aptamers (130 proteins, HR 0.79 (CD4) – 1.25 (CGA/FSHB), FDR < 0.05) (Fig. 2e), all of which overlapped with model 1 (Fig. 2f). When comparing the two models, 43% of the serum proteins remained significant after *APOE*-ε*4* adjustment, indicating that their LOAD association is independent of the *APOE*-ε*4* genotype (Table 1). Adjusting for additional AD risk factors and eGFR (see Methods) retained 38 significant LOAD-associated aptamers (35 proteins, HR 0.80 (CD4) – 1.26 (SMOC1), FDR < 0.05) (model 3, Supplementary Table 2), which may reflect specific processes affecting risk of LOAD but not driven by currently established risk factors.

As hazard ratio variability can arise with lengthy follow-up time, secondary analyses were implemented with a 10-year follow-up cut-off, which revealed mostly overlapping results (Supplementary Note 1, Supplementary Tables 3 and 4, Extended Data Fig. 1). We did however detect protein associations specific to the shorter follow-up time, which potentially reflect processes that take place closer to the LOAD diagnosis. As there may be further differences in proteomic profiles depending on whether protein sampling occurred before or after LOAD diagnosis, we additionally considered the protein profile of the 167 individuals with prevalent LOAD at baseline (Supplementary Note 2, Extended Data Fig. 2a-c, Supplementary Tables 5–7). Interestingly, many of the proteins associated with increased risk of incident LOAD showed the opposite direction of effect for prevalent LOAD, although generally not statistically significant (Extended Data Fig. 2d). These contrasting results suggest an important temporal element in the LOAD-associated proteome. In total there were 346 aptamers (329 unique proteins) associated with LOAD when all outcomes (incident and prevalent LOAD), follow-up times and models were considered (Supplementary Tables 2, 3, 5, 6, and 7).

To evaluate which biological processes are reflected by the overall incident LOAD-associated protein signature in AGES, we performed a gene set enrichment analysis (GSEA). The strongest enrichment for protein associations in model 1 was observed for gene ontology (GO) terms related to neuron development and morphogenesis (Fig. 2h–i, Supplementary Table 8). The proteins driving the enrichment included Neural Cell Adhesion Molecules 1 and 2 (NCAM1, and NCAM2), Netrin 1 (NTN1), Contactin 1 (CNTN1), Neuropilin 1 (NRP1), Fibronectin Leucine Rich Transmembrane Protein 2 (FLRT2), Matrix Metallopeptidase 2 (MMP2) and Cell Adhesion Molecule L1 like (CHL1). GSEA of the protein profiles of model 2, where *APOE*-ε*4* carrier status was adjusted for, showed similar enrichment results (Supplementary Table 8), demonstrating that these terms were mainly driven by the *APOE*-ε*4* independent component of the LOAD-associated protein profile.

### Serum proteins with APOE - dependent association to incident LOAD.

**Figure 3:**
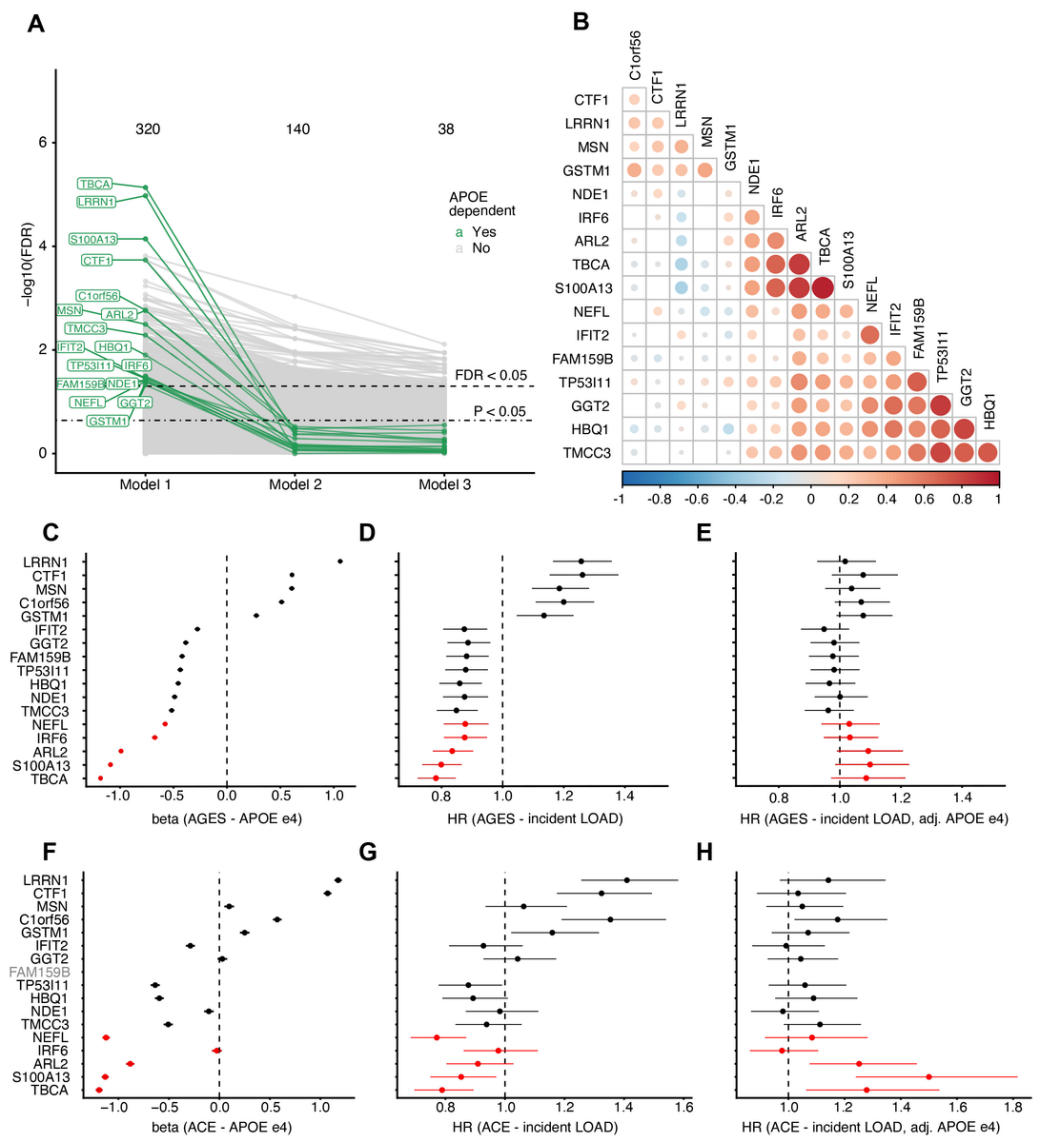
Proteins with *APOE*-ε*4* dependent association to incident LOAD in AGES (n = 5127). **a)** Spaghetti plot showing the statistical significance of protein associations with incident LOAD across the three Cox PH models, highlighting a set of 17 unique proteins (green) whose association with incident LOAD is attenuated upon *APOE*-ε*4* adjustment. The horizontal lines indicate FDR < 0.05 (dashed) and P < 0.05 (dot-dashed). **b)** Pairwise Pearson’s correlation between the 17 *APOE*-dependent proteins. **c)** Forest plot showing the linear associations between *APOE* genotype and the 17 *APOE*-dependent proteins. The beta coefficient shows the change in protein levels per ε4 allele count. **d-e)** Forest plots showing the associations between the 17 APOE-dependent proteins and incident LOAD d)without *APOE*-ε*4* adjustment (model 1) and **e)** with *APOE*-ε*4* adjustment (model 2). LOAD-HR indicates risk per SD increase of protein levels. Proteins that change direction of effect between the two models are highlighted in red.

As previously mentioned, 43% of the protein associations with incident LOAD were independent of *APOE*-ε*4*. Of the remaining 57% that were affected by *APOE*-ε*4* adjustment, we identified 17 proteins whose associations with incident LOAD were particularly strongly affected by *APOE*-ε*4* carrier status (Table 2, Fig. 3a, Extended Data Fig. 3, Supplementary Table 2). These proteins, hereafter referred to as *APOE*-dependent proteins, were defined as proteins significantly (FDR < 0.05) associated with incident LOAD in *model 1* but whose nominal significance was attenuated (P > 0.05) or direction of effect changed upon *APOE*-ε*4* adjustment in *model 2*. These *APOE*-dependent proteins included those with the strongest associations to LOAD prior to adjusting for the *APOE*-ε*4* allele (Fig. 2a, d). The levels of the APOE protein itself were not associated with either incident or prevalent LOAD. Figure 3b shows the intra-correlations among the 17 APOE-dependent proteins. All the 17 *APOE*-dependent proteins were strongly regulated by the *APOE*-ε*4* allele (Fig. 3c, Table 2, Extended Data Fig. 4, Supplementary Table 9), with the ε*4* allele increasing the levels of five of the proteins and decreasing the levels of the other 12. Accordingly, we observed that *increased* levels of the five *APOE*-ε*4* upregulated proteins and *decreased* levels of the 12 *APOE*-ε*4* downregulated proteins were also associated with higher risk of LOAD, yielding a hazard ratio above and below one, respectively (Fig. 3d). As per definition, most of the *APOE*-dependent proteins lost significance upon *APOE*-ε*4* adjustment yet interestingly, the direction of effect inverted for five proteins after *APOE*-ε*4* adjustment (ARL2, IRF6, NEFL, S100A13, TBCA) (Fig. 3e).

The HR conferred by *APOE*-ε*4* for incident LOAD in AGES was 2.1 (Cox PH P = 1.23e-27) per each ε*4* allele. To evaluate if any of the 17 *APOE*-dependent proteins might mediate the effect of *APOE*-ε*4* on incident LOAD, we considered the change in HR for *APOE*-ε*4* on risk of incident LOAD when adjusting for individual proteins. We found that adjustment for most proteins resulted in a minor effect decrease, suggesting they do not mediate the *APOE*-ε*4* effect on LOAD (Extended Data Fig. 5a). Intriguingly, however, the adjustment for four proteins (NEFL, ARL2, TBCA and S100A13) caused an increase of ~ 10% in *APOE*-ε*4* effect size (Extended Data Fig. 5a-b). Thus, the effect of *APOE*-ε*4* on LOAD is partly masked by secondary opposing associations between these proteins and LOAD, which are further explored below. The effect of *APOE*-ε*4* carrier status on LOAD risk was largely unchanged in a multivariable model containing all 17 *APOE*-dependent proteins, thus not supporting a mediating effect of these proteins for the LOAD risk conferred by *APOE*-ε*4* (base Cox PH model: HR = 2.1, P = 1.23e-27, multivariable Cox PH model: HR = 2.2, P = 3.2e−10). However, although not direct mediators, the 17 proteins could be blood-based readouts of a true mediator within tissue-specific pathological processes occurring prior to LOAD diagnosis.

**Figure 4:**
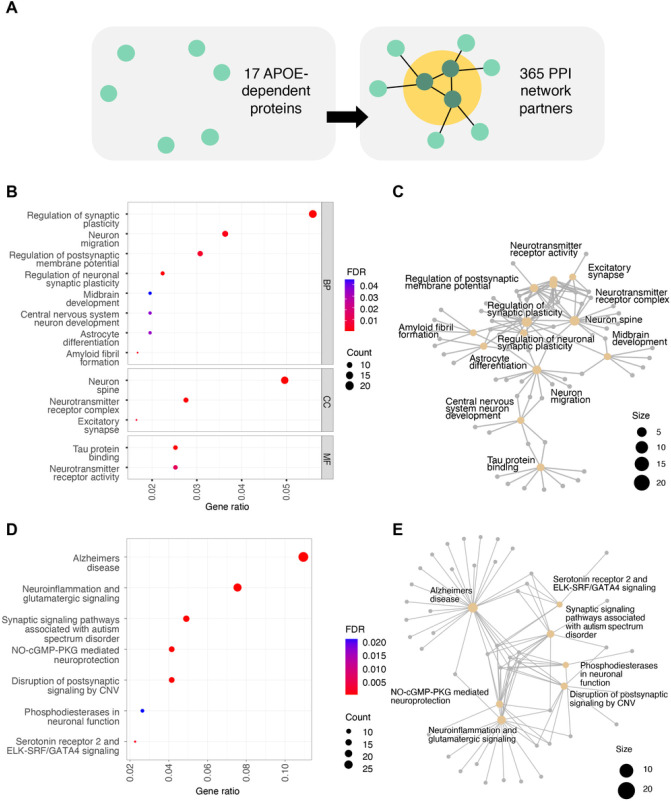
Functional enrichment analysis of *APOE*-dependent protein-protein interaction partners. **a)** A scheme of the PPI partners selection, where first degree partners of the *APOE*-dependent proteins were extracted from the InWeb database. **b-c)** Enrichment of selected Gene Ontology terms for the PPI partner proteins shown as **b)** dotplot and **c)** gene-concept network. **d-e)**Enrichment of top seven unique Wikipathways shown as **d)** dotplot and **e)**gene-concept network.

To map out potential tissues of origin for the circulating levels of the 17 *APOE*-dependent proteins, we considered gene expression data from the Human Protein Atlas^31^. We observed that five (LRRN1, TMCC3, FAM159B, NEFL, GSTM1) of the *APOE*-dependent proteins had elevated gene expression in brain compared to other tissues and additional two (IFIT2, NDE1) clustered with brain-specific genes (Table 2). Of the remaining *APOE*-dependent proteins, six were universally expressed, including in brain tissue, and four were enriched in other tissues. We did not detect any significantly enriched molecular signatures nor GO terms for the 17 *APOE*-dependent proteins (Supplementary Table 7). However, a network analysis of measured and inferred physical protein-protein interactions^32^ revealed that the *APOE*-dependent proteins interact directly with proteins involved in neuronal response- and development, neuroinflammation and AD (Fig. 4, Supplementary Table 10–12, Supplementary Note 3).

Given the well-established relationship between APOE and cholesterol^33^ we explored the potential effect of serum lipid levels on the association between LOAD and the 17 *APOE*-dependent proteins (Supplementary Table 13, Extended Data Figs. 6–7, Supplementary Note 4). Our findings suggest that, while many of the *APOE*-dependent proteins are associated with cholesterol levels, it is not the driver of their link to LOAD.

### External validation of protein associations with incident LOAD in the ACE cohort.

We set to externally evaluate our observations in an independent cohort, the ACE - Alzheimer Center Barcelona (n = 1,341), with SOMAscan platform (7k) measurements from plasma of individuals who were referred to the center. The longitudinal component of ACE consists of individuals who had been diagnosed with mild cognitive impairment (MCI) at the center and had been followed up. A total of 719 participants had follow-up information and 266 converted to LOAD over a median follow-up of 3.14 years (Supplementary Table 14). Despite the fundamentally different cohorts, with AGES being population-based and using the 5K SOMAscan platform and ACE being based on individuals with established symptoms and the 7K SOMAscan platform, we replicated 36 protein associations with LOAD at nominal significance (P < 0.05) in the smaller ACE cohort (Table 3, Fig. 2f–g). Of those, 30 proteins were nominally significant in model 1 with 97% being directionally consistent with the observations in AGES (Fig. 2f). In model 2, 21 proteins were nominally significant, 86% of which were directionally consistent (Fig. 2g). After multiple testing correction, seven proteins remained statistically significant (FDR < 0.05), all of which were directionally consistent (Table 3, Fig. 2f–g). Six were statistically significant (FDR < 0.05) in model 1 (NEFL, LRRN1, TBCA, CTF1, C1orf56 and TIMP4) and one in model 2 (S100A13) (Supplementary Table 9). Of all 332 tested aptamers, 213 (64%) were directionally consistent regardless of significance in model 1 (Two-sided exact binomial test P = 2.0e-05) and 202 (61%) were directionally consistent in model 2 (Two-sided exact binomial test P = 0.002), demonstrating an enrichment for consistency in direction of effect. The protein associations replicated in the ACE cohort are of particular interest as they represent potentially clinically relevant candidates for LOAD that are consistent in two different contexts, in both a general population and a clinically derived symptomatic sample set. However, our results suggest that many of the proteins that associate with long-term LOAD risk are not strongly associated with the conversion from MCI to AD, which is further into the AD trajectory and may also explain the limited overlap between the proteins associated with prevalent and incident LOAD in AGES.

### External validation of reversed LOAD association conditional on APOE-ε4 for a subset of proteins.

Specifically considering the *APOE*-dependent proteins, the association between the *APOE*-ε*4* allele and the proteins was replicated for 13 of 17 proteins in the ACE cohort (Fig. 3f). Furthermore, the change in direction of effect for incident LOAD upon *APOE*-ε*4* adjustment was replicated in the ACE cohort for 4 of 5 proteins (ARL2, NEFL, S100A13 and TBCA) (Fig. 3g–h) (Supplementary Table 9), with even larger effects observed in the ACE cohort compared to AGES in the *APOE*-ε*4* adjusted model and three proteins (ARL2, S100A13 and TBCA) becoming statistically significant (P < 0.05). Thus, the attenuation of the primary LOAD associations for these proteins upon *APOE*-ε*4* adjustment meet the criteria of *APOE*-ε*4* dependence (see Methods). No significant interaction between protein and *APOE*-ε*4* carrier status on AD risk was observed in either the AGES or ACE cohorts. Taken together, our results show that these proteins are strongly downregulated by *APOE*-ε*4*, and consequently show an inverse relationship with incident LOAD, but when adjusting for the *APOE*-ε*4* allele, their association to LOAD is still significant but reversed – suggesting a secondary non-*APOE*-ε*4*-mediated process affecting these same proteins in relation to LOAD in the opposite direction that is more strongly observed a cohort of individuals with MCI than in the population-based AGES cohort.

### Mendelian randomization to identify potential causal associations between proteins and LOAD.

The proteins associated with LOAD could include proteins causally related to the disease, or proteins whose serum level changes reflect a response to prodromal or genetic liability to LOAD. To test this hypothesis, we performed a bi-directional two-sample MR analysis, including the targets of all 346 aptamers associated with LOAD in our study. Genetic variant associations for serum protein levels were obtained from a catalog of cis-protein quantitative trait loci (pQTLs) from AGES^24^ while variant associations with LOAD were extracted from a recent GWAS on 39,106 clinically diagnosed LOAD cases, 46,828 proxy-LOAD and dementia cases and 401,577 controls of European ancestry^19^. In total, 117 (34%) of the LOAD-associated serum aptamers had cis-pQTLs that were suitable as genetic instruments and were included in the protein-LOAD MR analysis (Supplementary Table 15).

In the forward MR analysis, two proteins, integrin binding sialoprotein (IBSP) and amyloid precursor protein (APP), had support for causality (Supplementary Table 16). IBSP had a risk-increasing effect for LOAD in both the causal (OR = 1.26, FDR = 0.03) and observational analysis (incident LOAD full follow up, HR = 1.13, FDR = 0.04). APP had a protective effect for LOAD in both the causal (OR = 0.76, FDR = 0.03) and observational analysis (incident LOAD full follow up, HR = 0.87, FDR = 0.047). Notably, while not statistically significant, we observed suggestive support for a protective effect of genetically determined serum levels of acetylcholinesterase (ACHE, OR = 0.92, P = 0.061), a target of clinically used therapeutic agent for dementia^34^ (Supplementary Table 16, Extended Data Fig. 8). In a forward MR analysis of the *APOE*-dependent protein interaction partners, two proteins, APP and MAPK3, had support for causality (Supplementary Tables 10–12, Supplementary Note 3).

**Figure 5:**
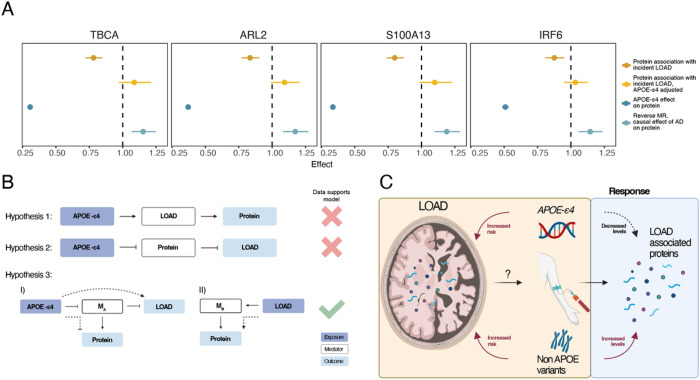
Reverse Mendelian randomization analysis. **a)** Comparison of hazard ratios for incident LOAD with and without *APOE*-ε*4* adjustment in the observational analysis (Cox PH), the effects of *APOE*-ε*4* on protein levels and reverse MR odds ratios (excluding the *APOE* locus) for the four APOE-dependent proteins that change direction of effect in both observational and causal analyses when *APOE* is accounted for. **b-c)**Visual summaries of the observed data. **b)** Mediation diagrams showing 3 possible hypotheses that could explain the relationship between *APOE*-ε*4*, LOAD and the four proteins shown in **a)**. Our analyses do not support the hypothesis that LOAD mediates the effect of *APOE*-ε*4* on proteins (Hypothesis 1) nor the other way around (Hypothesis 2). However, our results from both the observational and causal analyses support the hypothesis that two mechanisms are at play that affect the same proteins in the opposite direction (Hypothesis 3). **c)** The *APOE*-ε*4* mutation leads to increased risk of LOAD via its effects in brain tissue. The same mutation results in a downregulation of serum levels of four proteins that are themselves negatively associated with incident LOAD. Additionally, other non-*APOE* LOAD risk variants lead to upregulation of the same proteins in the reverse MR analysis, possibly reflecting a response to LOAD or its genetic liability.

As most of the observational protein associations in the current study were detected for incident LOAD, and thus reflect changes that take place before the onset of clinically diagnosed disease, it is unlikely that their levels and effects are direct downstream consequences of the disease after it reaches a clinical stage. However, they may reflect a response to a prodromal stage of the disease. We therefore performed a reverse MR to test if the observed changes in serum protein levels are likely to occur downstream of the genetic liability to LOAD, which may capture processes both at the prodromal and clinical stage. The *APOE* locus is likely to have a dominant pleiotropic effect in the reverse MR analysis (Supplementary Table 17, Extended Data Fig. 9, Supplementary Note 5), as it has a disproportionately strong effect on LOAD risk compared to all other common genetic variants, while also being a well-established pQTL trans-hotspot, affecting circulating levels of up to hundreds of proteins^24,25,27^. We therefore performed the primary reverse MR analysis using only LOAD-associated genetic variants outside of the *APOE* locus as instruments. We found two proteins (S100A13 and ARL2) that were significantly (FDR < 0.05) affected by LOAD or its genetic liability (Supplementary Table 17, Extended Data Figs. 9–10). Interestingly, both were among the 17 previously identified *APOE*-dependent LOAD proteins, together with two additional proteins that were nominally significant in the reverse MR (TBCA, P = 4.4e-4, FDR = 0.051 and IRF6, P = 7.9e-4, FDR = 0.055). Thus intriguingly, these findings suggest that these four proteins are upregulated by LOAD, in contrast to the observed *APOE*-ε*4* downregulation of the same proteins (Fig. 5). This supports our findings of competing biological effects described above (Fig. 3e, Extended Data Fig. 5) and collectively our results indicate that simultaneous opposing effects of *APOE*-ε*4* on one hand and LOAD on the other result in differential regulation of these proteins in serum (Fig. 5b).

We performed a replication analysis of the effect of *APOE*-ε*4* on protein levels and the reverse MR results for these four proteins using published protein GWAS summary statistics from two recent studies^25,35^. In the external datasets, the downregulation of all four proteins by *APOE*-ε*4* (as determined by the rs429358 C allele) was replicated. In the reverse MR analysis (excluding the *APOE* locus), the upregulation of protein levels by LOAD liability observed in AGES was also detected for two proteins (S100A13 and TBCA) in both validation cohorts, reaching significance (P < 0.05) in the study by Ferkingstad et al. (Extended Data Fig. 11, Supplementary Table 18). While the two proteins changed direction in a similar manner as in AGES, the effect size was considerably smaller in the validation cohorts. Importantly, however, individuals in these two cohorts are much younger than those in AGES, with mean ages of 55 and 48 years for the Ferkingstad et al. and Sun et al. studies, respectively, compared to 76 years in AGES. Therefore, we conducted an age-stratified reverse MR analysis in AGES, which showed a strong age-dependent effect, with a much larger effect of LOAD genetic liability on protein levels in individuals over 80 years old compared to those younger than 80 years (Extended Data Fig. 11). The effect size in AGES individuals under 80 years old was in line with the effect observed in the validation cohorts. Thus, if the upregulation of these proteins reflects a response to prodromal or preclinical LOAD, an older cohort may be needed to detect an association of the same degree as we found in AGES. However, the observed support in the validation cohorts for the discordant effects of *APOE* vs non-*APOE* LOAD-associated genetic variants on the same serum proteins strongly implicates these proteins as directly relevant to LOAD, potentially as readouts of biological processes that are both disrupted by *APOE*-ε*4* and modulated in the opposite manner as a response to genetic predisposition to LOAD or the disease onset in general.

Together, these results indicate that LOAD or its general genetic liability causally affects the levels of some *APOE*-dependent proteins, but this effect is simultaneously masked by the strong effects of the *APOE* locus in the other direction (Fig. 5a). These outcomes strengthen results described above, showing that the levels of these four proteins are strongly downregulated in *APOE*-ε*4* carriers and lower levels of these proteins are therefore associated with increased risk of LOAD in an *APOE*-dependent manner (Fig. 5b). Simultaneously, the reverse MR analysis shows that the collective effect of the other non-*APOE* LOAD risk variants is to upregulate the serum levels of these same proteins, possibly reflecting a response mechanism to LOAD pathogenesis (Fig. 5c). Again, this is in line with the observational analysis, where all four proteins changed direction of effect when adjusting for *APOE*-ε*4* (Fig. 5a, Fig. 2d–e).

### Overlap with the AD brain and CSF proteome.

**Figure 6:**
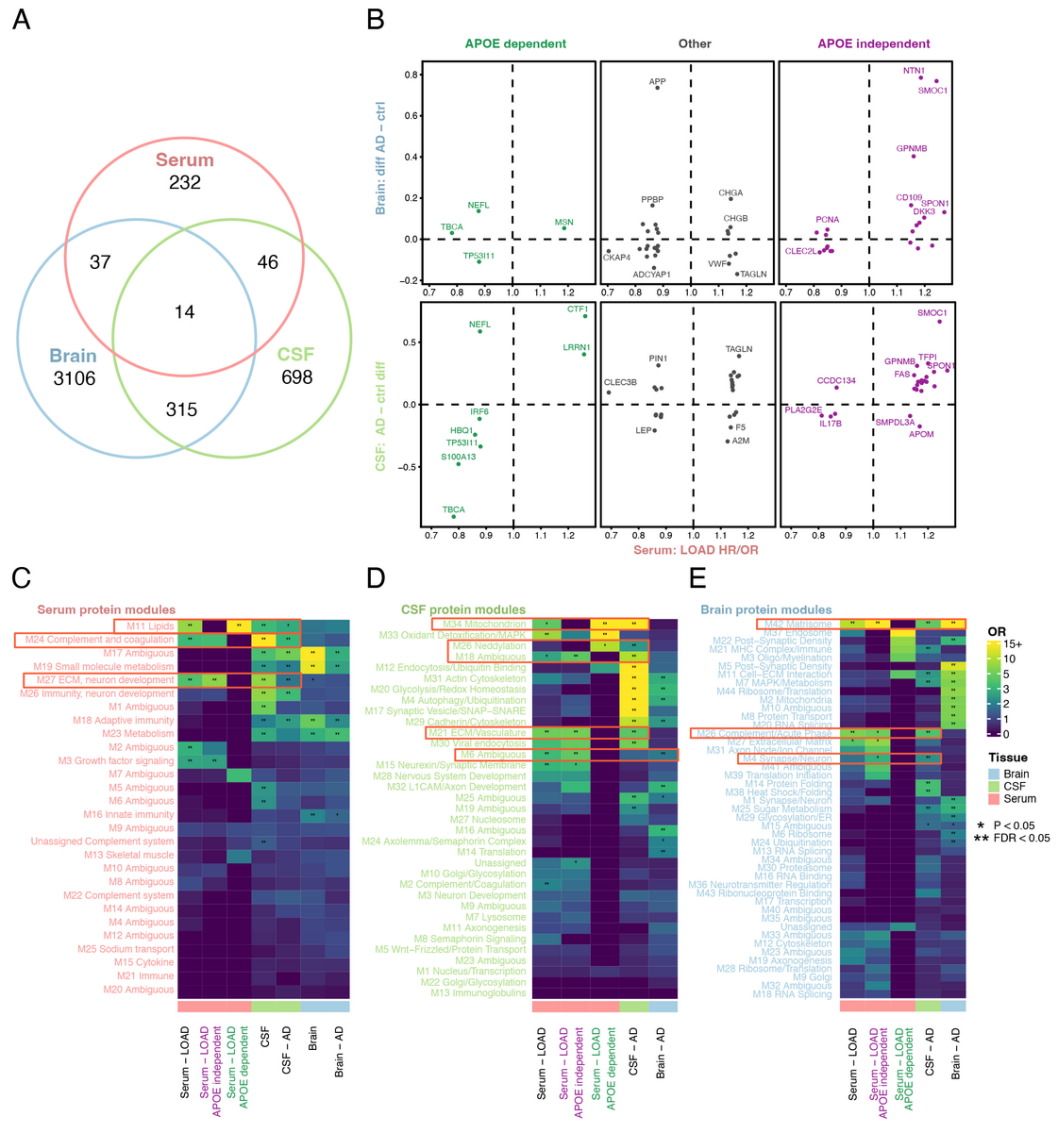
Overlap between AD protein signatures in serum, brain and CSF. **a)** A Venn diagram showing the overlap of AD-associated proteins in serum, brain and CSF. **b)** A comparison of the effect sizes for AD associated proteins that overlap between serum and brain (top) and serum and CSF (bottom). The proteins are stratified based on the *APOE*-dependence in AGES for incident LOAD. The effect size in AGES is shown for incident LOAD model 1 (Cox PH), except for proteins that were uniquely identified using the shorter 10-year follow-up (Cox PH) or prevalent LOAD (logistic regression), in which case the respective effect size from the significant association is shown. **c-e)** Heatmap showing the enrichment (Fisher’s test) of AD-associated proteins by tissue type (x-axis) in the AGES serum protein modules, **d)** Emory CSF protein modules and **e)** Emory brain protein modules (y-axis).

To evaluate to what extent our LOAD-associated serum proteins reflect the proteomic profile of AD in relevant tissues, we queried data from recent proteomic studies of AD in cerebrospinal fluid (CSF)^36^ and brain^37^ which also describe tissue specific co-regulatory modules. We observed that of our LOAD-associated serum proteins, 51 proteins were also associated with AD in brain as measured by mass-spectrometry, with 32 (63%) being directionally consistent (Fig. 6a–b) (Supplementary Tables 19–20). Higher directional consistency was observed within the *APOE*-independent protein group, or 15 (71%) of 21 proteins associated with AD in brain tissue. Additionally, 60 proteins were directly associated with AD in CSF as measured with SOMAscan (7k) (Fig. 6a) with 46 (77%) being directionally consistent (Fig. 6b)^21^. The proportion of directionally consistent associations between serum and CSF was higher in both the *APOE*-independent and dependent protein groups, or 88% (22 of 25 and 7 of 8 for *APOE*-independent and dependent proteins, respectively) (Fig. 6b, Supplementary Table 19). However, directional inconsistency between plasma and CSF AD proteomic profiles has been reported before in a similar comparison^38^. Fourteen proteins overlapped between all three tissues in the context of AD (Fig. 6a) (Supplementary Table 19). Many of these proteins have established links or are highly relevant to LOAD, such as Spondin 1 (SPON1), involved in the processing of amyloid precursor protein (APP)^39^; Secreted Modular Calcium-Binding Protein 1 (SMOC1) previously proposed as a biomarker of LOAD in postmortem brains and CSF^40^; Netrin-1 (NTN1), an interactor of APP and regulator of amyloid-beta production^41^; Neurofilament light (NEFL), previously proposed as a plasma biomarker for LOAD and axon injury^42,43^ and Von Willebrand factor (VWF), known for its role in blood clotting and associations with LOAD^44^ (Supplementary Table 19). Notably, some of the *APOE*-dependent proteins were associated with AD across all three tissues such as TBCA and TP53I11.

We have previously described the co-regulatory structure of the serum proteome, which can broadly be defined as 27 modules of correlated proteins^26^ (Supplementary Table 21). In the current study we found that among the 346 aptamers (329 proteins) associated with LOAD (prevalent or incident, any model), five serum protein modules (M27, M3, M11, M2 and M24) were overrepresented (Fig. 6c, Supplementary Table 22). In particular, the 140 *APOE*-independent aptamers were specifically overrepresented in module M27, enriched for proteins involved in neuron development and the extracellular matrix, and module M3 that is associated with growth factor signaling pathways (Supplementary Table 22). By contrast, the 17 *APOE*-dependent proteins were specifically enriched in protein module M11 (Supplementary Table 22), which is strongly enriched for lipid pathways and is under strong genetic control of the *APOE* locus^26^. Serum modules M27, M24 and M11 were all enriched for AD-associations in CSF (Fig. 6c). We next sought to understand to what extent our LOAD-associated proteins identified in serum might reflect AD protein signatures in CSF and brain tissue. Among the LOAD-associated proteins measured in serum, we found the *APOE*-dependent and *APOE*-independent proteins to be enriched in different CSF modules, most of which were also linked to AD (Fig. 6d, Supplementary Table 22). In brain tissue, the serum *APOE*-independent LOAD proteins were particularly enriched in brain module M42 (Matrisome), which is enriched for extracellular matrix (ECM) proteins^37^. Strikingly, M42 was strongly enriched for the AD-proteomic profiles of all three tissues (Fig. 6e, Supplementary Table 22). Interestingly, members of this module (SMOC1, APP, SPON1, NTN1, GPNMB) showed some of the strongest associations in serum to incident LOAD in our study (Fig. 2d, Supplementary Table 2) as well as in brain (Fig. 6b, Supplementary Table 22).

This module has furthermore been demonstrated to be correlated with amyloid beta (Aβ) deposition in the brain and some of its protein constituents (e.g MDK, NTN1 and SMOC1) have been shown to colocalize with and bind to Aβ^37^. Additionally, the *APOE* locus regulates M42 levels in the brain (mod-qTL), and while the APOE protein is a member of module M42, this regulation was found to not be solely driven through the levels of the APOE protein itself^37^. Our results simultaneously show that other members of the module, such as SPON1 and SMOC1, exhibit an *APOE*-independent association to incident LOAD in serum. Interestingly, these same two proteins are increased in CSF thirty years prior to symptom onset in autosomal dominant early onset AD^45^. In summary, we demonstrate significant overlaps in LOAD-associated protein expression across blood, CSF and brain on both an individual protein level and on protein module level.

## Discussion

**Figure 7:**
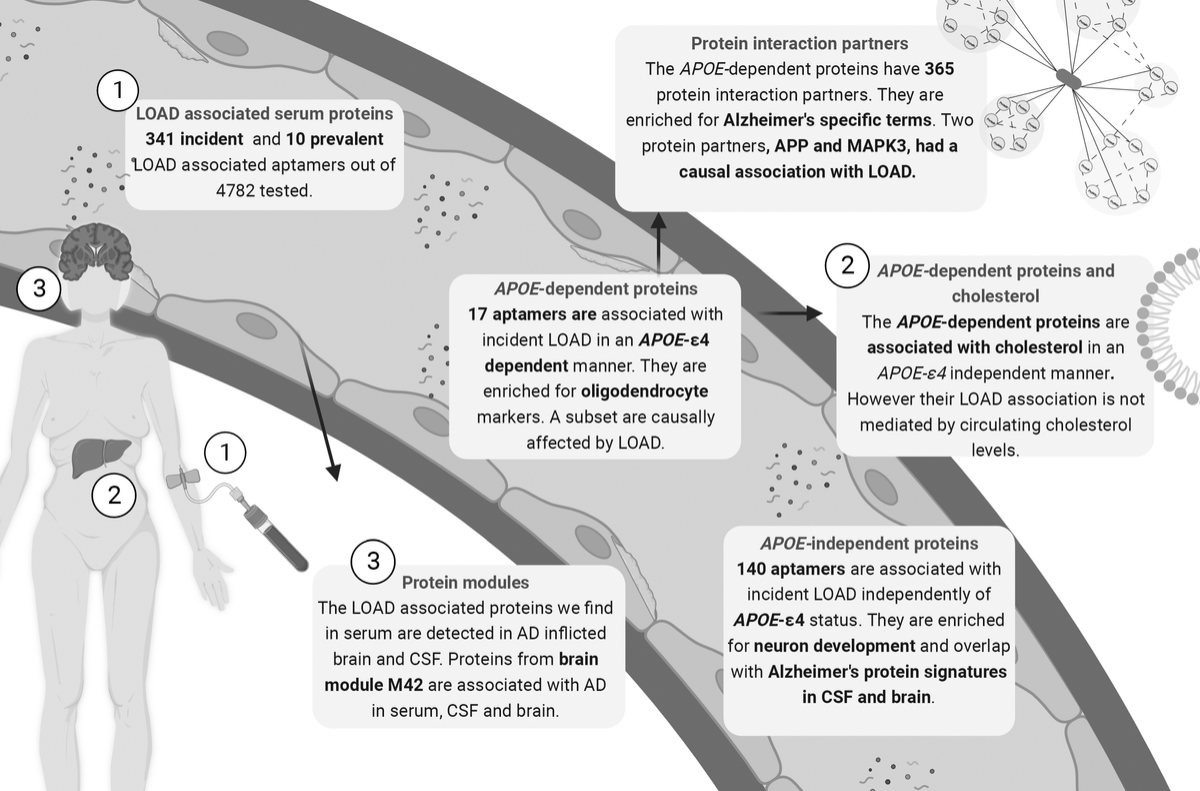
Graphical summary of the results.

We describe a comprehensive mapping of the serum protein profile of LOAD that provides insight into processes that are independent or dependent on the genetic control of *APOE*-ε*4* (Fig. 7). We identified 329 proteins in total that differed in the incident or prevalent LOAD cases compared to non-LOAD participants in a population-based cohort with long-term follow-up. Among these, we identified a novel grouping of proteins based on their primary LOAD-association being statistically independent of (130 proteins), or dependent on (17 proteins) *APOE*-ε*4* carrier status. Many of the *APOE*-independent proteins are implicated in neuronal pathways and are shared with the LOAD-associated CSF and brain proteome. The 17 *APOE*-dependent proteins overlap with AD-associated protein modules in CSF and interact directly with protein partners involved in LOAD, including APP. Another key finding is, amongst these 17 proteins, four proteins (ARL2, S100A13, TBCA and IRF6) change LOAD-associated direction of effect both observationally and genetically when taking *APOE*-ε*4* carrier status into account. Importantly, we replicate this directional change both observationally for three proteins (ARL2, S100A13, TBCA) and genetically for two proteins (S100A13, TBCA) in external cohorts. Collectively, our results suggest that while their primary association with LOAD reflects the risk conferred by *APOE*-ε*4*, there exists a secondary causal effect of LOAD itself on the protein levels in the reverse direction as supported by the MR analysis, possibly reflecting a response to the disease onset.

Previous studies identifying proteins associated with LOAD have been limited to a cross-sectional cohort or are based on all-cause dementia^18,46–48^. Here we extend those findings by distinguishing LOAD cases from other types of dementia in a prospective cohort study to identify LOAD-specific serum protein signatures preceding clinical onset. Furthermore, our comparative approach of statistical models with and without *APOE*-ε*4* adjustment provides a novel compartmentalized view of the LOAD serum protein profile and demonstrates how protein effects can differ depending on genetic confounders which are imperative to take into consideration. We found that the proteins associated with incident LOAD in our study, in particular those independently of *APOE*-ε*4* such as GPNMB, NTN1, SMOC1 and SPON1, overlap with the proteomic profile of LOAD in CSF^38^ and brain^37^, are enriched for neuronal pathways and have been functionally implicated with LOAD (Table 1), which may reflect an altered abundance of neuronal proteins in the circulation during the prodromal stage of LOAD. These overlaps that we find across independent cohorts and different proteomics technologies suggest that the serum levels of some proteins have a direct link to the biological systems involved in LOAD pathogenesis and may even provide a peripheral readout of neurodegenerative processes prior to clinical diagnosis of LOAD. In particular, the proteins that show directionally consistent effect sizes suggest exceptional AD-specific robustness as the measurements vary by tissue, methodology and populations.

We identified 17 proteins with a particularly strong *APOE*-dependent association to incident LOAD, of which eight were also associated with prevalent AD in CSF. The association between *APOE*-ε*4* and circulating levels of these proteins has been reported by our group^24,26,27^ and others^49^, but their direct association with incident LOAD has to our knowledge not been previously described. These *APOE*-dependent proteins may point directly to the processes through which *APOE*-ε*4* mediates its risk on LOAD and provide a readout of the pathogenic process in the circulation of the approximately 50% of LOAD patients worldwide carrying the variant^21,22^. While our data does not provide information on the tissue-origin of the *APOE*-dependent proteins, nine either exhibit brain-specific gene expression, cluster with brain-specific genes^50^ or have been associated with LOAD at the transcriptomic or protein level in brain tissue or CSF (Table 2). At the genetic level, a lookup in the GWAS catalog^51^ shows that an intron variant in the *IRF6* gene has a suggestive GWAS association with LOAD via *APOE*-ε*4* carrier status interaction^52^. In addition, variants in the *TMCC3* gene have been linked to LOAD^76^, educational attainment^53^ and caudate volume change rate^54^ and variants in the *TBCA* gene have been suggestively associated with reaction time^55^ and PHF-tau levels^56^. Collectively, the gene expression patterns for these proteins in the brain, interactions with proteins involved in neuronal processes and suggestive associations between genetic markers in or near these genes and brain-related outcomes suggest that these *APOE*-dependent proteins may reflect brain-specific processes affected by *APOE*-ε*4* carrier status that affect the risk of developing LOAD. Importantly, the association patterns for ARL2, S100A13 and TBCA suggest the presence of a pathway that is downregulated by *APOE*-ε*4* early in life, given the consistent effect of *APOE*-ε*4* on the same proteins in younger cohorts, but upregulated at the onset of LOAD, as supported by the larger observed effects in the *APOE*-ε*4* adjusted analysis in the ACE cohort of individuals who are closer to diagnosis on the AD trajectory than those in AGES. Additional studies are required to expand on these interpretations and dissect the complex mechanisms at play and to determine if the modulation of the process represented by these proteins has therapeutic potential.

Two proteins, IBSP and APP, were identified to potentially have a causal role in LOAD. IBSP was previously associated with plasma amyloid-β and incident dementia^57^, while APP is the precursor protein for amyloid-β^58^. Based on the MR analysis for the LOAD-associated proteins that could be tested, the majority do not appear to be causal in and of themselves but their association with incident LOAD may still reflect changes that occur years before the onset of LOAD that could be of interest to target before irreversible damage accumulates.

A major strength of this study is the high-quality data from a prospective longitudinal population-based cohort study with detailed follow-up, broad coverage of circulating proteins and a comprehensive comparison to the AD-proteome in CSF and brain. The limitations of our study include that our results are based on a Northern European cohort and cannot necessarily be transferred directly to other populations or ethnicities. Additionally, while we partly replicate our overall findings in an external cohort, a greater replication proportion could be anticipated in a more comparable cohort. The ACE cohort consists of clinically referred individuals with MCI and proteomic measurements performed on a different version of the SOMAscan platform. Additionally, different normalization procedures were applied by SomaLogic for the two SOMAscan versions, which may have an effect on the LOAD associations^48^. Further studies are required to determine the impact of time to event, platform and normalization approaches on the associations between circulating proteins and LOAD. Regardless of these differences, we did replicate the majority of the *APOE*-dependent LOAD associations, including the *APOE*-dependent change in effect for ARL2, S100A13 and TBCA. We could not test all LOAD-associated proteins for causality, including most of the APOE-dependent proteins, due to lack of significant cis-pQTLs for two thirds of the proteins, thus we cannot exclude the possibility that some could be causal but missed by our analysis. Finally, despite our LOAD diagnosis criteria it is possible that some of our findings reflect processes related to dementia in general. As a result, it is critical that these findings be validated in individuals with established amyloid-beta and tau deposits, as well as in experimental settings.

The proteins highlighted in this study and the mechanisms they point to may be used as a source of biomarkers or therapeutic targets that can be modulated for the prevention or treatment of LOAD. This large prospective cohort study, using both a longitudinal and cross-sectional design, represents a unified and comprehensive reference analysis with which past and future serum protein biomarkers and drug targets can be considered, compared, and evaluated.

## Methods

### AGES study population

Participants aged 66 through 96 were from the Age, Gene/Environment Susceptibility (AGES)-Reykjavik Study cohort. AGES is a single-center prospective population-based study of deeply phenotyped subjects (n = 5,764, mean age 76.6 ± 5.6 years) and survivors of the 40-year-long prospective Reykjavik study, an epidemiologic study aimed to understand aging in the context of gene/environment interaction by focusing on four biologic systems: vascular, neurocognitive (including sensory), musculoskeletal, and body composition/metabolism^30^. Of the AGES participants, 3,411 attended a 5-year follow-up visit. LOAD diagnosis at AGES baseline and follow-up visits was carried out using a three-step procedure described in Sigurdsson et al.^76^. Cognitive assessment was carried out on all participants. Neuropsychological testing was performed on individuals with suspected dementia. Individuals remaining suspect for dementia underwent further neurologic and proxy examinations in the second diagnosis step. Thirdly, a panel comprising of a neurologist, geriatrician, neuroradiologist, and neuropsychologist assessed the positive scoring participants according to international guidelines^30^ and gave a dementia diagnosis.

The participants were followed up for incident dementia through medical and nursing home reports and death certificates. The follow-up time was up to 16.9 years, with the last individual being diagnosed 16 years from baseline. Nursing home reports were based on intake exams upon entry or standardized procedures carried out in all Icelandic nursing homes^77^. The participants diagnosed at baseline were defined as prevalent LOAD cases while individuals diagnosed with LOAD during the follow-up period were defined as incident LOAD cases. All prevalent non-AD dementia cases (n = 163) were excluded from analyses.

Age, sex, education, and lifestyle variables were assessed via questionnaires at baseline. Education was categorized as primary, secondary, college, or university degree. Smoking was characterized as current, former, or never smoker. *APOE* genotyping was assessed via microplate array diagonal gel electrophoresis (MADGE)^78^. BMI and hypertension were assessed at baseline. BMI was calculated as weight (kg) divided by height (m) squared, and hypertension was defined as antihypertensive treatment or BP >140/90 mm Hg. Type 2 diabetes was defined from self-reported diabetes, diabetes medication use, or fasting plasma glucose ≥7 mmol/L. Serum creatinine was measured via the Roche Hitachi 912 instrument and estimated glomerular filtration rate (eGFR) derived with the four-variable MDRD study equation^79^. The AGES study was approved by the Nation Bioethics Committee (NBC) in Iceland (approval number VSN-00–063), and by the National Institute on Aging Intramural Institutional Review Board, and the Data Protection Authority in Iceland.

### Proteomic measurements

The proteomic measurements in AGES have been described in detail elsewhere^27,80^ and was available for 5,457 participants. Briefly, a custom version of the SOMAscan platform (Novartis V3–5K) was applied based on the slow-off rate modified aptamer (SOMAmer) protein profiling technology^81,82^ including 4,782 aptamers that bind to 4,137 human proteins. Serum was prepared using a standardized protocol^83^ from blood samples were collected after an overnight fast, stored in 0.5 ml aliquots at −80°C and serum samples that had not been previously thawed were used for the protein measurements. All samples were run as a single set at SomaLogic Inc. (Boulder, CO, US). Hybridization controls were used to adjust for systematic variability in detection and calibrator samples of three dilution sets (40%, 1%, and 0.005%) were included so that the degree of fluorescence was a quantitative reflection of protein concentration. All aptamers that passed quality control had median intra-assay and inter-assay coefficient of variation (CV) < 5%. Finally, intraplate median signal normalization was applied to individual samples by SomaLogic instead of normalization to an external reference of healthy individuals, as is done for later versions of the SOMAscan platform (https://somalogic.com/wp-content/uploads/2022/07/SL00000048_Rev-3_2022-01_-Data-Standardization-and-File-Specification-Technical-Note-v2.pdf).

Of the 37 *APOE*-independent and *APOE*-dependent proteins highlighted in Tables 1 and 2, respectively, orthogonal mass spectrometry (MS) has verified the specificity of 7 aptamers (6 proteins) in previous studies^26.^ Twelve additional aptamers were profiled (CD4 (3143_3_1), BRD4 (10043_31_3), SPON1 (5496_49_3), SMOC (13118_5_3), LRRN1 (11293_14_3), S100A13 (7223_60_3), CTF1 (13732_79_3), ARL2 (12587_65_3), C1orf56 (5744_12_3), MSN (5009_11_1), IRF6 (9999_1_3) and NEFL (10082_251_3)), and two additional aptamers (C1orf56 (5744_12_3) and MSN (5009_11_1)) were confirmed with SOMAmer pull down mass spectrometry (SP-MS) using elderly patient serum samples (>65 years) purchased from BioIVT (Table 2). The new confirmations’ methodology is consistent with previous publications^26^, but the instrumentation was updated. Data-dependent analysis was performed on an Orbitrap Eclipse operated in positive ionization mode, with electrospray voltage 1500V and ion transfer tube temperature of 275°C applied. Full MS scans with quadrupole isolation were acquired in the Orbitrap mass analyzer using a scan range of 375–1500 m/z, standard AGC target, and automatic maximum injection time. Data dependent scans were acquired in the Orbitrap with a 0.7 m/z quadrupole isolation window, 50,000 resolution, 50% normalized AGC target, 200 ms maximum injection time, and 38% HCD collision energy over a 2 sec cycle time. Dynamic exclusion of 45 sec relative to +/− 10ppm reference mass tolerance was applied. The peptides were eluted Aurora Ultimate 25cm × 75um ID, 1.7um C18 nano columns over a 90 min gradient on the Vanquish Neo UHPLC system (Thermo Fisher Scientific). Raw data files were processed in Proteome Discoverer v2.5 with SequestHT database search using a canonical human FASTA database (20528 sequences, updated 04/08/22).

### ACE cohort

ACE Alzheimer Center Barcelona was founded in 1995 and has collected and analyzed roughly 18,000 genetic samples, diagnosed over 8,000 patients, and participated in nearly 150 clinical trials to date. For more details, visit www.fundacioace.com/en. The syndromic diagnosis of all subjects of the ACE cohort was established by a multidisciplinary group of neurologists, neuropsychologists, and social workers. Healthy controls (HCs), including individuals with subjective cognitive decline (SCD) diagnosis, were assigned a Clinical Dementia Rating (CDR) of 0, and mild cognitive impairment (MCI) individuals a CDR of 0.5. For MCI diagnoses, the classification of López *et al.,* 2003, and Petersen’s criteria were used^84–87^. The 2011 National Institute on Aging and Alzheimer’s Association (NIA-AA) guidelines were used for AD diagnosis^88^. All ACE clinical protocols have been previously published^89–91^. Paired plasma and CSF samples^92^, following consensus recommendations, were stored at −80°C. A subset of ACE cohort was analyzed with the SOMAscan 7k proteomic platform^93^ (n = 1,370), (SomaLogic Inc., Boulder, CO, US). The proteomic data underwent standard quality control procedures at SomaLogic and was median normalized to reference using the Normalization by Maximum Likelihood (ANML) method (https://somalogic.com/wp-content/uploads/2022/07/SL00000048_Rev-3_2022-01_-Data-Standardization-and-File-Specification-Technical-Note-v2.pdf). Additionally, *APOE* genotyping was assessed using TaqMan genotyping assays for rs429358 and rs7412 SNPs (Thermo Fisher). Genotypes were furthermore extracted from the Axiom 815K Spanish Biobank Array (Thermo Fisher) performed by the Spanish National Center for Genotyping (CeGen, Santiago de Compostela, Spain).

### Statistical analysis

Protein measurement data was centered, scaled and Box-Cox transformed, and extreme outliers excluded as previously described^80^. Sample size was not predetermined by any statistical method but rather by available data. The associations of serum protein profiles with prevalent AD (n = 167) were examined cross-sectionally via logistic regression at baseline. The associations of serum protein profiles with incident LOAD (n = 655) were examined longitudinally via Cox proportional-hazards models. Participants who died or were diagnosed with incident non-AD dementia were censored at date of death or diagnosis. To account for hazard ratio variability which may arise with lengthy follow-ups, a secondary analysis using 10-year follow-up cut-off of incident LOAD was performed (n_LOAD_ = 432). To compare the fits of the two follow-up times and test for time-dependence of the coefficients we used anova and the survsplit function from the Survival R package^94^. For both prevalent and incident LOAD, we examined three covariate-adjusted models. The primary model (model 1) included the covariates sex and age. Model 2 included as an additional covariate the *APOE*-ε*4* allele count. The third model (model 3) included additional adjustment for cardiovascular risk factors, lifestyle, and kidney function (BMI, type 2 diabetes, education, hypertension, smoking history, eGFR) as they have been associated with risk of LOAD^95^. Benjamini-Hochberg false discovery rate (FDR) was used to account for multiple hypothesis testing. Analyses were conducted using R version 4.2.1. ACE SomaLogic proteomics data was similarly Box-Cox transformed and association analysis performed in the same manner as in AGES.

*APOE*-dependence criteria of the proteins were defined as serum proteins that met FDR significance of < 0.05 in association with incident LOAD in model 1, thus unadjusted for the *APOE*-ε*4* allele, but whose nominal significance was abolished upon *APOE*-ε*4* correction in model 2 (P > 0.05). Serum proteins that remained nominally significantly associated with incident LOAD (P < 0.05) upon *APOE*-ε*4* correction but changed direction of effect were also considered to meet the APOE dependence criteria, as a reversal of the effect indicates that the primary association is driven by *APOE*-ε*4*.

Functional enrichment analyses were performed using Over-Representation Analysis (ORA) and Gene Set Enrichment Analysis (GSEA) using the R packages ClusterProfiler and fgsea^96,97^. The association significance cut-off for inclusion in ORA was FDR < 0.05. Background for both methods was specified as all proteins tested from the analysis leading up to enrichment testing. The investigated gene sets were the following: Gene Ontology, Human Phenotype Ontology, KEGG, Wikipathways, Reactome, Pathway Interaction Database (PID), MicroRNA targets (MIRDB and Legacy), Transcription factor targets (GTRD and Legacy), ImmuneSigDB and the Vaccine response gene set^98^. Finally, we looked into tissue gene expression signatures via the same methods (ORA and GSEA) using data from GTEX^99^ and The Human Protein Atlas, where gene expression patterns across tissues were categorized in a similar manner as described by Uhlen et al^50^ and tissue-elevated expression considered as gene expression in any of the categories ‘tissue-specific’, ‘tissue-enriched’ or ‘group-enriched’. MinGSSize was set at 2 when investigating the LOAD-associated serum proteins directly. When investigating the *APOE*-dependent protein interaction partners, minGSSize was set to 15 and maxGSSize was set to 500. Overrepresentation of brain cell type markers among LOAD-associated proteins was tested using a Fisher’s exact test, with the SOMApanel protein set as background. Tissue specificity lookup for the top LOAD associated proteins was based on the Human Protein Atlas version 22 (https://v22.proteinatlas.org/). For the protein-protein interaction (PPI) network analysis, PPIs from InWeb^32^ (n = 14,448, after Entrez ID filtering) were used to obtain the first-degree interaction partners of the *APOE*-dependent proteins.

### Protein comparisons across serum, CSF and brain

To compare protein modules and AD associations across tissues, protein modules and protein associations to AD were obtained from brain^37^ and CSF^36^. The brain data, from the Banner Sun Health Research Institute^100^ and ROSMAP^101^, included TMT-MS-based quantitative proteomics for 106 controls, 200 asymptomatic AD cases and 182 AD cases. The CSF samples were collected under the auspices of the Emory Goizueta Alzheimer’s Disease Research Center (ADRC) and Emory Healthy Brain Study (EHBS)^36^. The cohort consisted of 140 healthy controls and 160 patients with AD as defined by the NIA research framework^102^. Protein measurements were performed using TMT-MS and SomaScan (7k). Only SomaLogic protein measurements were included in the comparison between CSF and serum, which were median normalized. Proteins were matched on SomaLogic aptamer ID when possible but otherwise by Entrez gene symbol. Overlaps between modules and AD-associated (FDR<0.05) proteins across tissues were evaluated with Fisher’s exact test.

### Mendelian randomization

A two-sample bi-directional Mendelian randomization (MR) analysis was performed to first evaluate the potential causal effects of serum protein levels on AD (forward MR), and second to evaluate the potential causal effects of AD or its genetic liability on serum protein levels (reverse MR). All aptamers significantly (FDR<0.05) associated with LOAD (incident or prevalent) were included in the MR analyses, or a total of 346 unique aptamers (Supplementary Tables 2, 3 and 5), of which 320 aptamers were significant in the full follow-up incident LOAD analysis (models 1–3), 106 aptamers were significant in the 10-year follow-up incident LOAD analysis (model 1–3) and ten aptamers were significant in the prevalent LOAD analysis (models 1–3). Genetic instruments for serum protein levels were obtained from a GWAS of serum protein levels in AGES^24^ and defined as follows. All variants within a 1 Mb (±500 kb) cis-window for the protein-encoding gene were obtained for a given aptamer. A cis-window-wide significance level Pb = 0.05/N, where N equals the number of SNPs within a given cis-window, was computed and variants within the cis window for each aptamer were clumped (r^2^ ≥ 0.2, P ≥ Pb). The effect of the genetic instruments for serum protein levels on LOAD risk was obtained from a GWAS on GWAS on 39,106 clinically diagnosed LOAD cases, 46,828 proxy-LOAD and dementia cases and 401,577 controls of European ancestry^19^. Genetic instruments for the serum protein levels not found in the LOAD GWAS data set were replaced by proxy SNPs (r^2^ > 0.8) when possible, to maximize SNP coverage. Genetic instruments for LOAD in the reverse causation MR analysis were obtained from the same LOAD GWAS^24^, where genome-wide significant variants were extracted (P < 5e-8) and clumped at a more stringent LD threshold (r2 ≥ 0.01) than for the protein instruments to limit overrepresentation of SNPs from any given locus across the genome. In the reverse causation MR analysis, cis variants (±500 kb) for the given protein were excluded from the analysis to avoid including pleiotropic instruments affecting the outcome (protein levels) through other mechanisms than the exposure (LOAD). A secondary reverse causation MR analysis was performed excluding any variants in the *APOE* locus (chr19:45,048,858–45,733,201). Causal estimate for each protein was obtained by the generalized weighted least squares (GWLS) method^103^, which accounts for correlation between instruments. Causality for proteins with single cis-acting variants was assessed with the Wald ratio estimator. For the reverse causation MR analysis, the inverse variance weighted method was applied due to a more stringent LD filtering of the instruments. Instrument heterogeneity was evaluated with Cochran’s Q test and horizontal pleiotropy with the MR Egger test.

## Figures and Tables

**Table 1 –:** A summary table of the top 20 significant APOE-independent proteins associated with incident LjOAD in AGES (n = 5127). The effect size (Hazard Ratio (Confidence Interval)) and level of significance (P, FDR) is shown for model 2 in the Cox PH, adjusting for age, sex and *AP0E-ε4*. The final column indicates if the aptamers have been orthogonally validated by mass-spectrometry (MS)^26^. The *APOE*-independency is defined as proteins remaining significantly (FDR< 0.05) associated with incident LOAD after *APOE*-ε*4* adjustment.

Aptamer ID	Entrez symbol	Tissue specificity	Tissue cluster	Functional evidence in LOAD	HR	P	FDR	MS validated
3143_3_1	CD4	Liver, Lymphoid tissue, Parathyroid gland	Immune cells		0.79 (0.73–0.87)	1.9E-07	9.3E-04	
10043_31_3	BRD4	Low	Non-specific	Mediates the transcriptional regulation underlying learning and memory. Downregulation in cell models increases amyloid beta^59,60^	0.82 (0.75–0.89)	1.4E-06	3.4E-03	
5496_49_3	SPON1	Gallbladder	Non-specific	Binds to the BACE1 cleavage site of APP and prohibits APP from being cleaved and released as the Aβ form^61^	1.24 (1.13–1.36)	3.6E-06	3.9E-03	
8242_9_3	CLEC2L	Brain, Parathyroid gland, Retina	Brain		0.83 (0.77–0.9)	5.7E-06	3.9E-03	
8463_2_3	SOD3	Choroid plexus	Ciliated cells		1.21 (1.11–1.31)	5.2E-06	3.9E-03	Yes
10980_11_3	ACHE	Brain, Skeletal muscle, Tongue	Striated muscle	A serine hydrolase that degrades acetylcholine (Ach) and terminates neurotransmission^62^	1.22 (1.12–1.33)	3.9E-06	3.9E-03	
2991_9_2	IL1R1	Low	Adipose tissue	The receptor for IL-1, which orchestrates inflammatory responses in the nervous system and is upregulated in AD^63^	1.23 (1.12–1.34)	4.7E-06	3.9E-03	
8520_8_3	ADAM30	Testis	Testis	Involved in APP degradation via sorting to lysosomes, targeted CTSD activation and then full-length APP recycling^64^	0.83 (0.77–0.9)	1.1E-05	6.0E-03	
2447_7_4	PLA2G2E	Lymphoid tissue	Immune cells		0.82 (0.75–0.89)	1.2E-05	6.0E-03	
3336_50_1	TFPI	Liver, Placenta	Liver, Placenta		1.2 (1.11–1.31)	1.3E-05	6.0E-03	Yes
11178.21 _3	SVEP1	Adipose tissue, Placenta	Adipose tissue		1.21 (1.11–1.33)	2.5E-05	0.011	Yes
11109_56_3	SVEP1	Adipose tissue, Placenta	Adipose tissue		1.21 (1.11–1.32)	3.3E-05	0.011	Yes
4304.18.2	LCORL	Low	Cerebellum		0.82 (0.75–0.9)	3.2E-05	0.011	
2247.20.11	PROK1	Ovary, Placenta, Testis	Fibroblasts		0.84 (0.77–0.91)	3.1E-05	0.011	
4498.62.2	NCAM1	Brain, Heart muscle	Heart	A substrate of BACE1, which is responsible for the cleavage of APP and formation of amyloid beta^65–67^	1.2 (1.1–1.3)	4.0E-05	0.011	Yes
13118.5.3	SMOC1	Brain, Liver	Liver	Enriched in amyloid plaques in both early onset Alzheimer’s disease and down syndrome^68^	1.21 (1.11–1.33)	3.9E-05	0.011	
5660.51.3	SOD3	Choroid plexus	Ciliated cells		1.19 (1.09–1.29)	4.0E-05	0.011	
8052.115.3	NLGN1	Brain, Retina	Cerebellum	A potential target of the synaptotoxic effect of amyloid-p (Aβ) oligomers and Aβ fibrils^69–70^	1.18 (1.09–1.28)	4.5E-05	0.011	
8009.121.3	SURF1	Low	Non-specific		1.19 (1.1–1.3)	4.3E-05	0.011	
6973_2_3	IGF2	Placenta	Placenta	Reverses memory and synaptic deficits in APP transgenic mice^71^	0.84 (0.77–0.92)	5.4E-05	0.012

**Table 2 –:** **A summary table of the 17 APOE-dependent LOAD associated proteins**, describing their tissue specificity in the Human Protein Atlas v22, results from the association analyses in AGES (n = 5127), references for previous functional associations with LOAD and whether the aptamers have been orthogonally validated via by mass-spectrometry (MS)^26^. The *APOE*-dependency is defined as being significant (FDR < 0.05) in model 1 and fully non-significant in model 2 (P > 0.05) or reversed effect for incident LOAD.

					Incident LOAD (Cox PH)				*APOE*-ε*4* (linear regression)				
					Model 1		Model 2						
Aptamer ID	Entrez Symbol	Tissue specificity	Tissue cluster	Functional evidence in LOAD	HR	FDR	HR	P	Beta	SE	P	FDR	MS validation
11293_14_3	LRRN1	Brain	Brain	A substrate of BACE1, which is responsible for the cleavage of APP and formation of amyloid beta^72^	1.258	1.E-05	1.018	0.711	1.061	0.023	< 1E-300	< 1E-300	
7223_60_3	S100A13	Low	Mitochondria		0.799	7.E-05	1.099	0.092	−1.090	0.021	< 1E-300	< 1E-300	Yes
12501_10_3	TBCA	Low	Vesicular transport		0.782	7.E-06	1.086	0.145	−1.183	0.020	< 1E-300	< 1E-300	Yes
13732_79_3	CTF1	Low	Fibroblasts, ECM	Improves memory and learning deficits in a transgenic mouse model of Alzheimer’s disease ^73^	1.262	2.E-04	1.077	0.147	0.611	0.021	1.E-171	5.E-171	
12587_65_3	ARL2	Low	Striated muscle		0.835	0.002	1.093	0.077	−0.991	0.022	< 1E-300	< 1E-300	
5744_12_3	C1orf56	Testis	Spermatogenesis		1.200	0.002	1.070	0.113	0.513	0.026	6.E-85	9.E-85	
5009_11_1	MSN	Low	Neutrophils, Inflammatory response	Highly expressed in microglia and has increased expression in the human AD brain ^74^	1.186	0.003	1.039	0.388	0.608	0.025	9.E-122	2.E-121	Yes
8922_4_3	TMCC3	Brain	Brain, Nervous system development		0.849	0.005	0.962	0.364	−0.517	0.026	4.E-86	7.E-86	
7965_25_3	HBQ1	Bone marrow	Neutrophils, Humoral immune response		0.860	0.013	0.966	0.423	−0.455	0.025	1.E-69	1.E-69	
9999_1_3	IRF6	Esophagus, Skin	Squamous epithelial cells		0.876	0.032	1.033	0.458	−0.675	0.025	4.E-153	1.E-152	
9853_3_3	IFIT2	Bone marrow	Brain & bone marrow, Chromatin organization		0.875	0.035	0.949	0.214	−0.275	0.026	2.E-26	2.E-26	
13022_20_3	TP53I11	Low	Adipose tissue, ECM organization		0.879	0.036	0.981	0.653	−0.436	0.026	1.E-60	1.E-60	
10635_33_3	FAM159B	Brain and other	Unknown function		0.882	0.039	0.977	0.591	−0.419	0.026	8.E-56	9.E-56	
9765_4_3	NDE1	Low	Brain, Nervous system development		0.876	0.039	1.001	0.985	−0.489	0.025	3.E-80	5.E-80	Yes
10082_251_3	NEFL	Brain	Brain, Neuronal signaling	Cytoskeletal protein specific for neurons and proposed biomarker of neuronal injury ^75^	0.878	0.041	1.031	0.516	−0.578	0.024	2.E-121	4.E-121	
7239_9_3	GSTM1	Brain	Liver, Metabolism		1.136	0.042	1.077	0.087	0.276	0.026	1.E-26	1.E-26	
6334_9_3	GGT2	Kidney, Thyroid	Kidney, Transmembrane transport		0.887	0.048	0.981	0.646	−0.384	0.026	2.E-47	3.E-47	

**Table 3 –:** Replication of the LOAD associated proteins from AGES (n = 5127) in the ACE cohort (n = 719). All proteins with nominal P< 0.05 in either model from the Cox PH are shown. P and FDR values < 0.05 are highlighted in bold.

			Model 1				Model 2			
Aptamer	Gene symbol	APOE proteins AGES	HR	95% Cl	P	FDR	HR	95% Cl	P	FDR
11293_14_3	LRRN1	Dependent	1.410	1.256–1.582	**5.4E-09**	**1.8E-06**	1.142	0.969–1.346	0.112	0.839
5744_12_3	C1orf56	Dependent	1.354	1.190–1.541	**4.3E-06**	**5.2E-04**	1.175	1.022–1.352	**0.024**	0.565
13732_79_3	CTF1	Dependent	1.324	1.174–1.494	**4.7E-06**	**5.2E-04**	1.034	0.888–1.205	0.666	0.958
10082_251_3	NEFL	Dependent	0.771	0.683–0.870	**2.5E-05**	**0.002**	1.084	0.917–1.282	0.346	0.958
12501_10_3	TBCA	Dependent	0.789	0.695–0.895	**2.3E-04**	**0.015**	1.279	1.063–1.538	**0.009**	0.336
6462_12_3	TIMP4	Independent	1.261	1.109–1.434	**4.0E-04**	**0.022**	1.236	1.087–1.405	**1.2E-03**	0.198
8819_3_3	IGFBP2	Independent	1.253	1.087–1.445	**0.002**	0.085	1.250	1.082–1.443	**0.002**	0.204
2570_72_5	IGFBP2	Independent	1.237	1.080–1.415	**0.002**	0.085	1.234	1.077–1.414	**0.002**	0.204
8469_41_3	IGFBP2	Independent	1.230	1.066–1.418	**0.004**	0.164	1.222	1.059–1.410	**0.006**	0.316
10978_39_3	GH2	Independent	1.182	1.043–1.340	**0.009**	0.298	1.131	0.998–1.281	0.053	0.751
2813_11_2	AGRP	Other	1.187	1.039–1.355	**0.011**	0.346	1.196	1.048–1.364	**0.008**	0.323
7100_31_3	CD2	Independent	1.166	1.031–1.317	**0.014**	0.353	1.102	0.975–1.246	0.121	0.839
6168_11_3	TP53	Other	1.190	1.034–1.369	**0.015**	0.353	1.127	0.976–1.302	0.104	0.839
4964_67_1	ERAP1	Independent	0.861	0.763–0.972	**0.015**	0.353	0.841	0.742–0.953	**0.007**	0.316
7223_60_3	S100A13	Dependent	0.853	0.749–0.971	**0.016**	0.353	1.500	1.240–1.816	**3.1E-05**	**0.010**
4930_21_1	STC1	Independent	1.173	1.024–1.343	**0.021**	0.391	1.174	1.024–1.346	**0.021**	0.565
9539_25_3	COL26A1	Other	0.865	0.764–0.979	**0.022**	0.391	0.890	0.788–1.005	0.059	0.756
7239_9_3	GSTM1	Dependent	1.159	1.021–1.316	**0.023**	0.391	1.070	0.941–1.217	0.304	0.951
4929_55_1	SHBG	Other	1.168	1.022–1.335	**0.023**	0.391	1.149	1.005–1.313	**0.042**	0.751
13118_5_3	SMOC1	Independent	1.172	1.022–1.345	**0.024**	0.391	1.157	1.006–1.331	**0.041**	0.751
5660_51_3	SOD3	Independent	1.162	1.018–1.326	**0.026**	0.411	1.162	1.018–1.325	**0.026**	0.565
5581_28_3	FGL1	Other	1.149	1.015–1.301	**0.028**	0.430	1.118	0.988–1.266	0.077	0.757
13022_20_3	TP53I11	Dependent	0.877	0.777–0.991	**0.035**	0.458	1.059	0.930–1.206	0.388	0.958
8235_48_3	CHGB	Other	1.151	1.010–1.311	**0.035**	0.458	1.123	0.983–1.282	0.088	0.797
7016_12_3	GCNT1	Independent	0.864	0.754–0.990	**0.036**	0.458	0.874	0.763–1.001	0.052	0.751
13731_14_3	C7	Independent	1.159	1.010–1.331	**0.036**	0.458	1.147	1.001–1.316	**0.049**	0.751
2925_9_1	SERPINE1	Other	0.875	0.770–0.995	**0.042**	0.511	0.870	0.762–0.992	**0.038**	0.751
3290_50_2	CD109	Independent	0.884	0.784–0.996	**0.043**	0.511	0.896	0.794–1.012	0.076	0.757
6626_81_3	CHST12	Independent	0.871	0.761–0.997	**0.045**	0.518	0.904	0.790–1.036	0.146	0.848
6604_59_3	NDNF	Other	0.879	0.774–0.999	**0.048**	0.528	0.897	0.790–1.019	0.096	0.817
14129_1_3	IFNA7	Other	1.131	1.000–1.279	0.051	0.543	1.134	1.003–1.282	**0.045**	0.751
8072_19_3	MZT1	Independent	1.118	0.996–1.256	0.058	0.604	1.146	1.020–1.287	**0.022**	0.565
4982_54_1	PI3	Other	1.139	0.989–1.311	0.070	0.608	1.185	1.027–1.366	**0.020**	0.565
4297_62_3	SPON1	Independent	1.127	0.989–1.285	0.073	0.608	1.167	1.019–1.336	**0.025**	0.565
8242_9_3	CLEC2L	Independent	1.102	0.974–1.248	0.124	0.686	1.136	1.003–1.287	**0.045**	0.751
12587_65_3	ARL2	Dependent	0.909	0.804–1.028	0.130	0.686	1.252	1.075–1.457	**0.004**	0.255

## Data Availability

The custom-design Novartis SOMAscan is available through a collaboration agreement with the Novartis Institutes for Biomedical Research (lori.jennings@novartis.com). Data from the AGES-Reykjavik study are available through collaboration (AGES_data_request@hjarta.is) under a data usage agreement with the IHA. All other data supporting the conclusions of the paper are presented in the main text and freely available as a supplement to this manuscript.
